# Risk Factors for Headache Disorder in Patients With Unruptured Intracranial Aneurysms

**DOI:** 10.7759/cureus.38385

**Published:** 2023-05-01

**Authors:** Aureliana Toma, Rafael De La Garza Ramos, David J Altschul

**Affiliations:** 1 Neurological Surgery, Albert Einstein College of Medicine, Bronx, USA

**Keywords:** endovascular treatment, vascular disorder, cerebrovascular, headache disorder, aneurysm

## Abstract

Objective: Headache disorders are a prevalent yet frequently underestimated issue in patients with unruptured intracranial aneurysms (UIAs). The primary aim of this study is to systematically examine the incidence, specific characteristics, and associated risk factors of headache disorders in the context of individuals diagnosed with UIAs. Through this investigation, we hope to contribute valuable insights to the current understanding of this complex relationship and potentially inform future diagnostic and treatment approaches.

Methods: Data from 146 consecutive patients harboring UIAs were evaluated. The location and morphological characteristics of the aneurysm were analyzed. Factors associated with headache incidence and methods of treatment were investigated. The headache pattern in 48 patients was assessed using self-reported questionnaires.

Results: A total of 146 patients were identified. Out of 146 patients, 95 (65%) were in the Headache Group (HG) and 51 (35%) were asymptomatic and in the No Headache Group (NHG). Factors associated with a higher likelihood of headache were past or current tobacco, alcohol, and illicit drug use (p=0.029). On average, patients had 1.49 (SD=1) aneurysms in the HG and 1.43 (SD=.92) in the NHG group, respectively. In our series, the size of aneurysms, the status of the aneurysm (treated vs untreated), and the method of treatment did not significantly differ between the groups. There was a high incidence of headaches in patients with aneurysms of the ophthalmic segment (C6) of the internal carotid artery (ICA) and sphenoidal segment (M1) of the middle cerebral artery (MCA). Of 48 patients that completed headache questionnaires, 25 had headaches on more than 15 days a month. The majority of participants (85.4%) reported the severity of their pain as being greater than 5 on a scale of 10, while one-third (33.3%) experienced the maximum pain level of 10 out of 10.

Conclusion: Headache more often occurs in patients with aneurysms of the ophthalmic segment (C6) of the ICA and sphenoidal segment (M1) of the MCA. Its distinctive features are deep pain for more than 15 days a month. Although the treatment of aneurysms reduces the risk of aneurysmal rupture, its efficacy in relieving the headache is still uncertain.

## Introduction

Unruptured intracranial aneurysms (UIAs) represent a significant health concern as they can lead to severe complications such as subarachnoid hemorrhage (SH), which carries high morbidity and mortality rates. The clinical manifestations of UIAs are diverse and often non-specific, making their timely diagnosis and management challenging.

Headache is a common symptom in the general population, with primary headache disorders accounting for the majority of cases [1]. However, secondary headache disorders, which are caused by underlying intracranial conditions, can also occur and be associated with UIAs [2]. The relationship between headaches and UIAs is of particular interest, as understanding this association could contribute to improved diagnostic accuracy and more effective patient management.

Previous studies have reported the prevalence of headaches in patients with UIAs, with estimates ranging from 16.2% to 18% [3,4]. Headaches in these patients can present with varying characteristics such as intensity, duration, and location, and may be accompanied by additional features like aneurysm thrombosis [4] or microbleeds, both of which can be considered signs of impending SH [5]. Timely diagnosis is critical, as not all patients with unstable aneurysms experience the typical thunderclap headache; some may present with migraine-like headaches [5,6]. Despite advancements in imaging techniques and minimally invasive treatment modalities for aneurysms, the relationship between headaches and UIAs remains unclear. Several factors such as aneurysm size, location, and morphology, have been proposed to influence the occurrence and presentation of headaches in patients with UIAs, but the exact mechanisms and risk factors are not yet fully understood.

In this paper, we aim to provide a comprehensive overview of the current knowledge regarding the association between headache disorders and UIAs. We evaluate the possible correlations between aneurysm characteristics and the presence of headache disorders, identify and assess a wide range of risk factors and predictors in patients with unruptured intracranial aneurysms, and discuss the potential implications of our findings for the diagnosis and management of these patients.

## Materials and methods

Study design and inclusion criteria

Approval was obtained from the Institutional Review Board (IRB) of Albert Einstein College of Medicine in January 2019 to conduct this study (Approval number: 2019-10214). We evaluated data from 146 patients with a primary diagnosis of UIAs who were consulted by one neurosurgeon at a single medical center between January 2019 and January 2020. In this context, the term "prospective" refers to the patients who were identified and enrolled during the one-year study period, as opposed to being identified retrospectively from medical records. Patients who underwent surgical clipping of the aneurysm or had a craniotomy in the last 12 months from the day of the interview were excluded from the study.

Collected parameters

Collected data included demographic information, medical history, symptoms leading to aneurysm diagnosis, aneurysm laterality, size, location, and method of treatment. The size, location, and number of aneurysms were examined angiographically. Types of headaches were classified according to The International Classification of Headache Disorder 3rd edition (ICHD-III). Headache was characterized based on the severity on depth, feeling, time sequence, duration, and onset. We utilized the Visual Analogue Scale (VAS) for pain assessment, which is a widely accepted and validated tool for measuring pain intensity. The VAS is a continuous scale ranging from 0 to 10, with 0 indicating no pain and 10 representing the worst pain imaginable. Eligible patients were also asked to complete additional provided questionnaires related to their headache - The Hamilton-Veal Headache Questionnaire (HVHQ), and the 12-item Allodynia Symptom Checklist (ASC-12). 

Statistical analysis

Descriptive statistics were used to describe the study population. Two groups were established - the Headache Group (HG) and the No Headache Group (NHG). Comparisons were made via t-test, chi-square test, or analysis of variance (ANOVA) as appropriate. A univariate analysis was performed to identify factors associated with headache occurrence. For all analyses, p-values of <0.05 were considered significant. Data analysis was conducted in Microsoft Excel (version 16.4) and SPSS version 26 (IBM Corp., Armonk, NY).

## Results

Study population

A total of 146 patients were identified. Out of 146 patients, 95 (65%) had headaches and 51 (35%) were asymptomatic (Table 1). The mean age of our study population was 61 years (SD 12.8 years), and 111 patients (76%) were women. Patients in the HG were younger (59 versus 66 years; p<0.001) and less likely to be hypertensive (p=0.047), hyperlipidemic (p=0.001), and diabetic (p=0.029). Patients with headaches were significantly more likely to have a history of illicit drug use (p=0.029).

**Table 1:** Baseline characteristics of patients with unruptured cerebral aneurysms with and without headaches. †- represents the percent of patients with dizziness only. SAH: subarachnoid hemorrhage.

Variables	Total	No headache group	Headache group	P value
	N=146	N= 51	N= 95	
Age, mean (SD)	61 (12.8)	66 (13.2)	59 (11.9)	<0.001
Female, n (%)	111 (76)	35 (68.6)	76 (80.0)	0.125
Vascular risk factors				
Hypertension, n (%)	87 (59.6)	36 (70.6)	51(53.7)	0.047*
Hyperlipidemia, n (%)	49 (33.6)	26 (51)	23 (24.4)	0.001*
Diabetes, n (%)	29 (19.9)	15 (29.4)	14 (14.7)	0.034*
Smoking, n (%)	60 (41.1)	16 (31.4)	44 (46.3)	0.126
Alcohol, n (%)	37 (25.3)	12 (23.5)	25 (26.3)	0.712
Illicit drugs, n (%)	23 (15.8)	3 (5.9)	20 (21.1)	0.029*
Circumstances the aneurysm was identified				
Headache/Dizziness work-up, n (%)	53 (39.8)	10 (20.8) †	43 (50.6)	<0.001*
Change in quality of headaches, n (%)	7 (5.3)	0 (0)	7 (8.2)	
Stroke work-up	17 (12.8)	4 (8.3)	13 (15.3)	
Incidental, n (%)	15 (11.3)	12 (25)	3 (3.5)	
Past medical history of SAH	20 (13.7)	5 (9.8)	15 (15.8)	0.316

There was a significant difference between groups in circumstances that led the aneurysm to be identified (p<0.001). The leading cause to visit a doctor’s office in more than half of patients with headaches was the onset of a new headache and dizziness (50.6%) or change in quality of their headache (8.2%). Overall, 20 out of 146 patients (13.7%) had a past medical history of subarachnoid hemorrhage, without significant statistical difference between the HG and NHG. 

Aneurysm characteristics in patients with and without headaches are presented in Table 2.

**Table 2:** Aneurysm characteristics in patients with and without headaches. ICA: internal carotid artery; MCA: middle cerebral artery; ACA: anterior cerebral artery; PcomA: posterior communicating artery; AcomA: anterior communicating artery; PICA: posterior inferior cerebellar artery; SD: standard deviation.

Aneurysm’s characteristics	Total	No headache group	Headache group	P value
		N=51	N=95	
Number of aneurysms identified, mean (SD)		1.43 (.92)	1.49 (1.0)	0.751
Left side, n (%)	66 (50.0)	24 (51.1)	42 (49.4)	0.856
Vessel:				
· ICA	68 (47.6)	24 (47.1)	44 (47.8)	0.418
· ACA	10 (7.0)	5 (9.8)	5 (5.5)	
· MCA	28 (19.6)	12 (23.5)	16 (17.4)	
· PcomA	14 (9.8)	3 (5.9)	11 (12)	
· AcomA	11 (7.7)	4 (7.8)	7 (7.6)	
· Vertebral	3 (2.1)	2 (3.9)	1 (1.1)	
· PICA	3 (2.1)	0 (0)	3 (3.3)	
Arterial segment, n (%):				
· C4	17 (16.5)	8 (19.5)	9 (14.5)	.033*
· C5	12 (11.7)	5 (12.2)	7 (11.3)	
· C6	29 (28.2)	7 (17)	22 (35.5)	
· C7	10 (9.7)	5 (12.2)	5 (8.1)	
· A1	3 (2.9)	3 (7.3)	0 (0)	
· A2	3 (2.9)	0 (0)	3 (4.8)	
· M1	10 (9.7)	2 (4.9)	8 (12.9)	
· M2	14 (13.6)	7 (17.1)	7 (11.3)	
· M3	2 (1.9)	1 (2.4)	1.(1.6)	
· V4	3 (2.9)	3. (7.3)	0 (0)	
Aneurysm dimensions (mm)				
					Neck, mean (SD)	3.61 (2.1)	4.3 (2.8)	3.8 (1.7)	0.201
Width, mean (SD)	5.5 (2.25)	5.6 (1.9)	5.8 (2.4)	0.16					
					Height, mean (SD)	3.96 (2.87)	4.2 (2.07)	4.4 (3.2)	0.253
Height/Neck ratio, mean (SD)	1.18 (0.84)	1.2 (0.49)	1.3 (0.97)	.027*					
					Width/Neck ratio, mean (SD)	1.26 (0.57)	0.93 (0.29)	1.5 (0.57)	0.147
Underwent aneurysm treatment, n (%)	78 (53.4)	24 (47.1)	54 (56.8)	0.259
Method of treatment				
Clip, n (%)	8 (5.48)	0	8 (14.8)	
Endovascular, n (%)	70 (47.9)	24 (100)	46 (85.1)	.047*

On average, patients had 1.49 (SD=1) aneurysms in the HG and 1.43 (SD=.92) in the NHG group, respectively. There was a significant difference in the location based on the arterial segment (p=0.033). The aneurysm size and method of treatment did not significantly differ between the groups. 

Headache characteristics

Of the 95 patients with headaches, 48 agreed to complete the ICHD, HVHQ, and ASC-12 questionnaires to assess a more detailed description of their headaches (Table 3). The majority of respondents were female (85.4%) and over half of them reported having headaches for over 15 days per month (52.4%). The most common headache type was migraine-like in 54.2% of cases. Among patients who mentioned that their headache started after undergoing treatment for their aneurysm (22.9%), three patients underwent coil embolization, five underwent flow-diversion, one underwent WEB embolization, one underwent stent-assisted coiling, and one underwent surgical clipping. 

**Table 3:** Overall descriptive analysis of headaches pattern in patients with UIAs (total 48). †- Percent of subjects on drug therapy for pain management that achieved some degree of pain relief SAH: subarachnoid hemorrhage; UIAs: unruptured intracranial aneurysms.

Variable	Count	Percent
			Total patients with headache	48	
Female	41	85.4
Headaches >15 days/month	25	52.1
Past medical history of SAH	8	16.7
Onset of headache after treatment	11	22.9
Type of headache:		
· Migraine	26	54.2
· Tension Type headache	16	33.3
· other	6	12.5
Pain on a scale 1-10:		
· <5	7	14.6
· >5	41	85.4
*33.3% of patients reported 10 points pain		
Drug therapy for pain management	30	75
Drug therapy effectiveness	23	76.6 †
History of head injury	18	37.5
Family history of headaches	22	45.8

About one-third of patients reported severity of pain being 10/10 on a scale from 0-10 (33.3%). Seventeen patients (35.4%) experienced headaches ipsilateral to their aneurysm location. Of the 48 respondents, 22 (45.8%) had a family history of headaches and 18 (37.5%) had a history of traumatic brain injury. 

More detailed characteristics of headaches in patients with UIAs are summarized in Table 4.

**Table 4:** Headache characteristics in patients with unruptured aneurysms (total n=48).

Variable	Count	Percent
Headache depth:		
· Near surface	13	27.1
· Deep ache	29	60.4
· Both	4	8.3
Severity:		
· Mild	3	6.3
· Moderate	15	31.3
· Severe	15	31.3
· Extreme	14	29.2
Nature of pain:		
· Throbbing	27	56.3
· Burning	8	16.7
· Dull ache	15	31.3
· Knife like	16	33.3
· Neck ache	20	41.7
Time sequence:		
· Continuous	13	27.1
· Comes and goes	19	39.6
· Short time only	8	16.7
· Steady but builds up	6	12.5
· All of the above	1	2.1
Onset of headache:		
· Abrupt	26	54.2
· Gradual	19	39.6
Frequency of headache:		
· Every day	16	33.3
· Once/week	3	6.2
· Once/month	8	16.7
· Other	21	43.7

Patients with aneurysms of ACA, MCA, PCA, Acom, and Pcom, were more likely to have throbbing quality of headaches than patients with aneurysms of the ICA (p=0.01). Unruptured intracranial aneurysm of ICA was significantly associated with higher frequency of headache symptoms (every day) compared to headache frequency in other aneurysmal locations (p<0.001). 

## Discussion

Headache disorder remains a common and disabling symptom for many patients with brain aneurysms. Though generally asymptomatic, it is estimated that one-third of patients with UIAs experience some type of headache disorder [7]. Headache in patients with UIAs leads to constant anxiety and frustration and has a huge impact on quality of life [8,9]. In this study, we retrospectively reviewed patients with UIAs treated at our institution to identify the prevalence, characteristics, and factors associated with headaches.

Patients within the headache group were younger and more likely to have a history of illicit drug abuse. Previous studies have described the correlation of cocaine-related headaches [10,11]. It has been suggested that pathophysiological mechanisms inflicted in headache syndrome caused by cocaine consumption are oxidative stress, endothelial dysfunction, raised platelet activation, and raised production and activation of prostaglandins [10]. Furthermore, neuro stimulants like cocaine, amphetamine, and ecstasy have been implicated in the formation [12,13] and rapid growth of multiple intracerebral aneurysms [13,14] and are associated with a higher risk for aneurysmal rupture in the young population [15-18]. 

We found that patients that harbor cerebral aneurysms of the ICA at the level of C6 and of the MCA at the level of M1 are more likely to have headaches as a symptom. These findings may suggest that close proximity of the aneurysm to the dura mater, which is innervated along the middle cranial fossa, may be the cause of pain in these patients [19,20]. This assertion can be further substantiated by the observation that one-third of patients who completed the questionnaires experienced headaches on the same side as the aneurysm location. Another mechanism that may have an impact on producing a diffuse headache is total cerebral vasodilatation evoked by mechanical stimulation of meningeal sensory fibers at the base of the skull and subsequently, release of neuropeptides, that also play a role in headache pathogenesis [21]. 

We also reviewed the status and method of treatment, an important factor that might be associated with the prevalence of headaches in patients with symptomatic UIAs. Headache associated with the existence of aneurysm is depicted in the ICHD-III classification but reported data from previous investigations on how headache is affected by specific methods of treatments is limited and controversial. Previous studies reported dramatic post-procedure headache relief in patients treated surgically or with coil embolization [20,22-25]. Nevertheless, some studies suggest that endovascular treatment itself predisposes to the worsening of preexisting headaches or even leads to new onset of headaches [26-29]. In our study, we did not find any significant difference in the modality of treatment of the aneurysm and the incidence of headaches between HG and NHG. Additionally, no significant differences were observed in aneurysmal sidedness or size. 

This review assessed a wide range of metrics including aneurysm location, number of aneurysms, modality of treatment, and detailed information about the headache pattern. The findings of our analysis challenge the widely-held belief that headaches are unlikely to be associated with unruptured intracranial aneurysms (UIAs) [24]. Contrary to this prevailing assumption, our study indicates that there is indeed a connection between certain types of aneurysms and headache disorders. This highlights the need for further research to better understand the relationship between UIAs and headaches, which could ultimately contribute to improved diagnosis and management strategies for patients with UIAs. The most obvious difference between our study and others is the inclusion of patients that underwent diverse methods of treatment and the extent to which we focused on detailed headache characteristics. Furthermore, our results provide additional evidence to support that patients with severe, deep ache headaches on more than 15 days per month, require further investigations, and when feasible, undergo aneurysmal treatment. 

Limitations

There are some limitations of this study. One limitation of our study is the high percentage of patients who declined to complete the headache questionnaire. This may have introduced selection bias and potentially affected the prevalence and risk factor estimates reported in our study. Future research should aim to minimize non-participation and explore strategies to increase response rates in order to obtain more representative samples and improve the accuracy of findings. Second, our study design included only a one-time discussion with patients and does not represent the dynamic state of headache syndromes pre- and postoperatively. Another limitation of our study is the lack of multivariable analysis, which could have helped identify independent risk factors and account for potential confounding variables. Future research with larger sample sizes and more comprehensive data collection may benefit from employing multivariable analysis to provide more robust insights into the relationships between aneurysm characteristics and headache disorders.

## Conclusions

Headache disorders occur more often in relatively younger patients with a history of tobacco, ethanol, or illicit drug use. Aneurysms of the ophthalmic segment (C6) of the ICA and sphenoidal segment (M1) of the MCA are more likely to cause headaches in patients with UIAs. Aneurysmal headache has distinctive features such as deep pain for more than 15 days a month with a severity of 10 out of 10. Although the treatment of aneurysms reduces the risk of subarachnoid hemorrhage, its efficacy in relieving headaches is still uncertain.
